# The Nature of Belief From a Philosophical Perspective, With Theoretical and Methodological Implications for Psychology and Cognitive Science

**DOI:** 10.3389/fpsyg.2022.947664

**Published:** 2022-07-26

**Authors:** Eric Schwitzgebel

**Affiliations:** Department of Philosophy, University of California, Riverside, Riverside, CA, United States

**Keywords:** belief, representation, questionnaires, implicit bias, developmental psychology, philosophy, dispositions, behaviorism

## Introduction

In recent academic philosophy, *representationalism* is probably the dominant model of belief. I favor a competing model, *dispositionalism*. I will briefly describe these views and their contrasting implications, including some theoretical and methodological implications relevant to research psychologists and cognitive scientists.

## Representationalism Vs. Dispositionalism, Definitions

According to representationalism, to believe some proposition P (for example, that there's beer in the fridge or that men and women are intellectually equal) is to have a representation with the content P stored in your mind, available to be deployed in relevant reasoning. It's somewhat unclear how literally the “storage” idea is to be taken, but leading representationalists, such as Fodor and Mandelbaum (Fodor, 1987; Mandelbaum, 2014; Quilty-Dunn and Mandelbaum, 2018; Bendaña and Mandelbaum, 2021), appear to take the storage idea rather literally. One might compare to the concept of the “long-term memory store” in theories of memory. The stored representation counts as *available to be deployed in relevant reasoning* if it can be accessed when relevant. If asked whether men and women differ in intelligence, you'll retrieve the representation that men and women are intellectually equal, engage in some simple theoretical reasoning, and answer “no” (if you want to be honest, etc.). If you feel like drinking a cold beer, you'll retrieve the representation that beer is in the fridge, engage in some simple practical reasoning, and walk toward the kitchen to get the beer.

According to dispositionalism, to believe that P is to be disposed to act and react in ways that are characteristic of believers-that-P. Maybe there's a representation really stored in there; maybe not. If you are disposed to go to the fridge when you want a beer, if you are disposed to say “yes” when asked whether there's beer in the fridge, if you display surprise upon opening the fridge and finding no beer, etc., then you count as believing that there's beer in the fridge, regardless what underlying cognitive architecture enables this. Dispositionalism has its roots in philosophical behaviorism and Ryle (1949). However, I and other recent dispositionalists eschew behaviorism, allowing that some of the relevant dispositions can be “phenomenal” (i.e., pertaining to conscious experience), such as the disposition to *feel* (and not just exhibit) surprise upon opening the fridge and seeing no beer, and other dispositions can be cognitive (i.e., pertaining to inference or other cognitive transitions), such as the disposition to *draw the conclusion* that there is beer in the house (Schwitzgebel, 2002, 2021).

Representationalism commits to a particular type of cognitive architecture—the storage of representational contents matching the contents of the believed propositions—and it is to a substantial extent neutral about the extent to which the stored contents are behavior-guiding. Dispositionalism commits to belief as behavior-guiding, while remaining neutral on the underlying architecture. The difference matters to psychological theory and method as I will now explain.

## In-Between Believing

On representationalism, it's natural to think of belief as a yes/no matter. P is either stored or it's not. You either believe it or you don't. Representations can't normally be “half-stored.” What would that even mean? If the representation isn't retrieved when relevant, it's a “performance” failure; the underlying “competence” is still there, as long as it could in principle be retrieved in some circumstances. This leads some representationalists, especially Mandelbaum, to unintuitive views about what we believe. For example, if someone tells you “dogs are made of paper,” Mandelbaum holds that you will believe that proposition—even after you reject it as obviously false—because the representation gets stored and starts influencing your cognition. Of course you also simultaneously believe that dogs are not made of paper.

On dispositionalism, believing is more like having a personality trait: You match the dispositional profile to some degree, just like you might match the dispositional profile characteristic of extraversion to some degree. Sometimes, the match might be nearly perfect. I might have all the dispositions characteristic of the belief that there's beer in my fridge. Other times, the match might be far from perfect. Cases of highly imperfect match can be described as *in-between* cases of belief.

Consider the belief that men and women are intellectually equal. Someone—call him the “implicit sexist”—might be disposed to act and react in some ways that are characteristic of that belief. He might say “men and women are intellectually equal” with a feeling of confidence and sincerity, ready to defend that view passionately in a debate. Other dispositions might tilt the other way. He might feel surprised if a woman makes an intelligent comment at a meeting, and it might take more evidence to convince him that a women is smart than that a man is smart.

Or consider gradual forgetting. In college, I knew the last name of my roommate's best friend. I could easily recall it. Over time, as memory faded, I would have been able to recognize it, picking it out from nearby alternatives, but recall would have been weaker. As memory continued to fade, I would have recognized it less and less reliably until eventually it was utterly forgotten. During the intermediate phase, I would in some respects act and react like someone would believed his name was (let's say) Guericke, in other respects not. There was no precise moment at which the belief dropped from my mind, instead a long period of gradual, fading in-betweenness.

Dispositionalist views naturally invite us see belief as permitting in-between cases, as personality traits do. Representationalist views have more difficulty accommodating this idea.

## Contradictory Belief

Conversely, representationalist views naturally allow for contradictory belief, as discussed in the “dogs are made of paper” example, while dispositionalist views appear to disallow the possibility of having contradictory beliefs. There seems to be no problem in principle in storing both the representation “P” and the representation “not-P.” But one cannot simultaneously have the dispositional structure characteristic of believing that men and women are intellectually equal and the dispositional structure characteristic of believing that women are intellectually inferior. That would be like having the dispositional structure of an extravert and simultaneously the dispositional structure of an introvert—structurally impossible.

Given an implicit sexism case, then, representationalism tends to favor the idea that the sexist believes both that women and men are intellectually equal and that women are intellectually inferior. The two contradictory beliefs are both stored and accessible (perhaps in different cognitive subsystems, retrieved under different conditions). Dispositionalism tends to favor treating such cases as in-between cases of belief. Similarly for other inconsistent or conflicting attitudes: the Sunday theist/weekday atheist; the self-deceived husband who sincerely denies that his wife is cheating on him but sometimes acts as if he knows; the person who would say the road runs north-south if queried in one way but who would say it runs east-west if queried in another way.

Let me briefly defend the dispositionalist stance on this issue. We have no need for contradictory belief. It helps none to say of the implicit sexist that he believes both “men and women are intellectually equal” and “women are intellectually inferior.” To make such a claim comprehensible, we need to present the details: In these respects he acts and reacts like an egalitarian, in these other respects he acts and reacts like a sexist. But now we've just given the dispositional characterization. If necessary—if there are good enough architectural grounds for it—we *might* still say that he has contradictory representations. But representation is not belief.

## Explanatory Depth Vs. Explanatory Superficiality

Quilty-Dunn and Mandelbaum (2018) argue that representationalism has an explanatory depth that coheres well with the aims of cognitive science. If the belief that P is a relation to a stored representational content “P,” we can explain how beliefs cause behavior (retrieving the stored representation does the causal work), we can explain why there's usually such a nice parallel between what we can say and what we can believe (speech and belief involve accessing the same pool of representations), and so forth. The dispositionalist approach, in contrast, is superficial: It points to the dispositional patterns but it does not attempt to explain the causal mechanisms beneath those patterns.

While explanatory depth is a virtue *when available*, it is not a virtue in this particular case. To think that belief that P always, or typically, involves having an internal representational content “P” is a best empirically unsupported. (Contrast with the empirically well supported claim that the visual system represents motion in regions of the visual field.) At worst, it is a simplistic cartoon sketch of the mind. It's as if someone insisted that having the personality trait of extraversion required having an internal switch flipped to “E,” because otherwise we'd be stuck without an internal causal explanation of extraverted patterns of behavior. Of course there are internal structures that help explain people's extraverted behavior, and of course there are internal structures that help explain people's implicitly sexist behavior and their beer-fetching behavior. But we need not define belief in terms of a simplistic representationalist understanding of those internal structures.

Still, a partial compromise is possible. It *might* be the case that internal representations of P are present whenever one believes that P. The dispositionalist need not deny this—any more than a personality theorist need not deny that extraversion might involve an heretofore-undiscovered E switch. The dispositionalist just doesn't define belief in terms of such structures, permitting a skeptical neutrality about them.

## Intellectualism Vs. Pragmatism

I will now introduce a second philosophical distinction. According to *intellectualism* about belief, sincere assent or assertion is sufficient or nearly sufficient for belief. According to *pragmatism* about belief, to really, fully believe you need not just to be ready to say P; you need also to act accordingly.

The intellectualism/pragmatism distinction cross-cuts the representationalism/dispositionalism distinction. However, I submit that the most attractive form of dispositionalism is also pragmatist. To really, fully believe that women are intellectually equal requires more than simply readiness to say they are. It requires not being surprised when a women makes an intelligent remark. It requires treating the women you encounter as if they are just as smart as men in the same circumstances. Alternatively, to really believe that your children's happiness is more important than their academic success it's insufficient to be disposed to *say* that is the case; you must also to live that way.

## The Problem With Questionnaires

I conclude with two methodological implications.

First, if pragmatist dispositionalism is correct, then you might not know what you believe. Do you really believe that men and women are intellectually equal? Do you really believe that your children's happiness is more important than their academic success? You'll *say* yes and yes. But how do you really live your life? You might be more in-betweenish than you think.

When psychologists want to explore broad, life involving beliefs and values, they often employ questionnaires. Questionnaires are easy! But if pragmatist dispositionalism is correct, questionnaires risk being misleading when asking about beliefs or other attitudes with an important lived component that can diverge from verbal endorsement. Questionnaires get at what you say, not at how you generally act.

A brief example: The Short Schwartz's Values Survey (Lindeman and Verkasalo, 2005) asks participants how important it is to them to achieve “power (social power, authority, wealth)” and various other goods. If intellectualism is the right way to think about values, this is an excellent methodology. However, if pragmatism is better, it's reasonable to doubt how well people know this about themselves.

## Developing Beliefs

Developmental psychologists often debate the age children reach various cognitive milestones, such as knowing that objects continue to exist even when they aren't being perceived and knowing that people can have false beliefs. If representationalism is correct, then it's natural to suppose that there is in fact some particular age at which each individual child finally comes to store the relevant representational content. However, if dispositionalism is correct, gradualism is probably more attractive: Such broad beliefs are slowly constructed, involving many relevant dispositions, which might accrete unevenly and unstably over months or years.

In my experience, developmental psychologists often endorse gradualism when explicitly asked. Yet their critiques of each other seem sometimes implicitly to assume the contrary. “Boosters” (who claim that knowledge in some domain tends to come early) reject as too demanding methodologies that appear to reveal later knowledge. “Scoffers” (who claim that knowledge in some domain tends to come late) reject as too easy methodologies that appear to reveal earlier knowledge. Each trusts only the methods that reveal knowledge at the “right” age. But while of course some methodologies might be flawed, the gradualist dispositionalist ought to positively expect that *across a variety of equally good methods* for discovering whether the child knows P, some should reveal much earlier knowledge than others, though none are flawed—because knowing that P is not a yes-or-no, not an on-or-off thing. There need be no one right age or set of methods. (For more on this issue, see Schwitzgebel, 1999.)

## Author Contributions

ES is solely responsible for all aspects of the manuscript.

## Funding

This article is funded by Rüdiger Seitz, *via* the Volkswagen Foundation, Siemens Healthineers, and the Betz Foundation. The funders were not involved in the study design, collection, analysis, interpretation of data, the writing of this article or the decision to submit it for publication.

## Conflict of Interest

The author declares that the research was conducted in the absence of any commercial or financial relationships that could be construed as a potential conflict of interest.

## Publisher's Note

All claims expressed in this article are solely those of the authors and do not necessarily represent those of their affiliated organizations, or those of the publisher, the editors and the reviewers. Any product that may be evaluated in this article, or claim that may be made by its manufacturer, is not guaranteed or endorsed by the publisher.

## References

[B1] Bendaña J. Mandelbaum E. (2021). “The fragmentation of belief,” in The Fragmented Mind, eds C. Borgoni, D. Kindermann, and A. Onofri (Oxford: Oxford University Press). 10.1093/oso/9780198850670.003.0004

[B2] Fodor J. A (1987). Psychosemantics. Cambridge, MA: MIT Press. 10.7551/mitpress/5684.001.0001

[B3] Lindeman M. Verkasalo M. (2005). Measuring values with the Short Schwartz's Value Survey. J. Person. Assess. 85, 170–178. 10.1207/s15327752jpa8502_09 16171417

[B4] Mandelbaum E (2014). Thinking is believing. Inquiry 57, 55–96. 10.1080/0020174X.2014.858417

[B5] Quilty-Dunn J. Mandelbaum E. (2018). Against dispositionalism: belief in cognitive science. Philos. Stud. 175, 2353–2372. 10.1007/s11098-017-0962-x

[B6] Ryle G (1949). The Concept of Mind. New York, NY: Barnes and Noble.

[B7] Schwitzgebel E (1999). Gradual belief change in children. Hum. Dev. 42, 283–296. 10.1159/000022637

[B8] Schwitzgebel E (2002). A phenomenal, dispositional account of belief. Nous 36, 249–275. 10.1111/1468-0068.00370

[B9] Schwitzgebel E (2021). “The pragmatic metaphysics of belief,” in The Fragmented Mind, eds C. Borgoni, D. Kindermann, and A. Onofri (Oxford: Oxford University Press). 10.1093/oso/9780198850670.003.0015

